# Some Metropolitan, Provincial, Scotch, and Irish Special Hospitals Compared

**Published:** 1893-11-04

**Authors:** 


### The Institutional Workshop.

### HOSPITAL ADMINISTRATION.

### IX.—SOME METROPOLITAN, PROVINCIAL, SCOTCH, AND IRISH SPECIAL HOSPITALS COMPARED.

Cost of Management.*

The interest excited in this series of articles on the cost of management of various hospitals has induced us to add an additional one to the number. The article which appeared at page 45 of The Hospital, October 21st, on Provincial, Scotch, and Irish Hospitals, states fully the essential differences between metropolitan and provincial institutions, so far as the raising of funds and the general management and expenditure are concerned. It is not necessary, therefore, to deal with these points again to-day. It will be seen from the table which follows that it contains particulars of five children's hospitals, of which three are situated in Birmingham, Bristol, and Manchester respectively, one in Edinburgh, and one in Glasgow. We have also included the Eye Infirmary at Glasgow and the Rotunda Lying-In Hospital at Dublin, because these are the two most important extra-metropolitan special hospitals to be met with in this country. We are of opinion that as far as children's hospitals are concerned they excite a very special interest amongst the residents in the towns where they are situated. They are essentially institutions in which ladies take a deep personal interest, and we know no more successful canvassers than members of the fair sex. Hence the cost of management should certainly be reasonably low, and we consider 15 per cent. to be the maximum sum, so far as this class of institution is concerned.

Those who are interested in the comparisons we are about to make should refer to the third article of the series, which deals with children's hospitals, and which appeared in our issue of September 9th, at page 381. It will be seen that we there fixed the maximum percentage of the cost of administration to that of management in a children's hospital in London at 15 per cent. also. We may add that the maximum cost of administration to that of maintenance in the case of lying-in hospitals is taken to be 20 per cent., and in the case of ophthalmic hospitals 15 per cent., as may be seen by referring to the articles which appeared in our issues of September 23rd (page 413) and of September 30th (page 429).

Children's Hospitals.

It is well to bear in mind that when a children's hospital is situated in the heart of a poor neighbourhood, far away from the public gaze, it would, perhaps, be justified in expending 20 per cent. on management, for the reasons set forth in the third article of this series, published September 9th last. Turning to the table, we have five children's hospitals to deal with, the largest containing 140 beds, of which 116 were constantly occupied, and the smallest 64 beds, of which 60 were constantly occupied. In what follows our readers must bear in mind that in the case of the Glasgow and Bristol Hospitals for Children the number of out-patients is given in brackets, it being possible in each case to entirely exclude the expenditure on the out-patient department, which has accordingly been ignored in calculating the percentages, so that they relate entirely to the cost of administration to maintenance of the hospital proper. It is remarkable that the Glasgow Hospital for Children with 70 beds should only cost 8·858 per cent., whereas the Bristol Hospital with 102 beds cost 21·293 per cent. Apparently this enormous difference is not due to any special feature peculiar to either institution, as the calculations have been made upon an identifical basis and are confined entirely to the expenditure of the hospital proper. The Edinburgh Hospital for Sick Children cost 19·279 per cent. to administer, whilst Manchester spent 13·734 per cent., and Birmingham 12·343 per cent. upon management. Most people will be struck with the relatively high expenditure at Bristol and Edinburgh, especially when the latter institution is compared with that at Glasgow. We think some explanation is called for in these cases, and we shall be very glad to publish anything on the subject that the respective committees may send us in due course.

Let us now compare these provincial and Scotch hospitals with the metropolitan children's hospitals.

Provincial, Scotch, and Irish Special Hospitals.

| Name of Hospital. | Beds for Occupation. | Average Number Occupied. | Total In-patients Treated. | Total Out-patients Admitted. | Percentage of Cost of Administration to that of Maintenance. | Percentage of Cost of Administration to Total Ordinary Expenditure. |
|---|---|---|---|---|---|---|
| Children. | | | | | | |
| Birmingham and Midland Free Hospital for Sick Children ... ... | 74 | 62 | 857 | 14,239 | 12·343 | 10·987 |
| Bristol Hospital for Sick Children ... | 102 | 93 | 882 | [3,964] | 21·293 | 17·555 |
| Edinburgh Royal Hospital for Sick Children ... ... ... ... | 64 | 60 | 523 | 9,069 | 19·279 | 16·162 |
| Glasgow Hospital for Sick Children | 70 | 66 | 654 | [6,337] | 8·858 | 8·137 |
| Manchester General Hospital and Dispensary for Sick Children ... | 140 | 116 | 1,296 | 9,531 | 13·734 | 12·076 |
| Lying-in. | | | | | | |
| Rotunda, Dublin ... ... ... | 90 | 59 | 2,145 | 6,168 | 9·179 | 8·407 |
| Eye. | | | | | | |
| Glasgow Eye Infirmary ... ... | 104 | 68 | 1,326 | 14,599 | 10·874 | 9·808 |

* Previous articles of this series have appeared in The Hospital, page 333, for August 19th; page 349 for August 26th; page 381 for September 9th; page 413 for September 23rd; page 429 for September 30th; page 13 for October 7th; page 29 for October 14th; and page 45 for October 21st, 1893.

Manchester may be fairly compared with the Hospital for Sick Children in Great Ormond Street, as although the latter contains 182 beds against 140 at the former institution, the number of beds constantly occupied at Great Ormond Street was 120 against 116 such beds at Manchester. Manchester treated 1,296 in-patients and 9,531 out-patients against 1,518 in-patients and 21,045 out-patients treated by the Great Ormond Street Hospital for Sick Children, the percentage of cost of administration to that of maintenance being 14·349 per cent. at Great Ormond Street against 13·734 per cent. at Manchester. It thus appears that the London institution expends upwards of one-half per cent. more than the provincial institution, whereas the percentage of cost of administration to total ordinary expenditure is 12·559 at the Hospital for Sick Children, against 12·976 at Manchester. Let us take another example. The Bristol Children's Hospital contained 102 beds, of which 93 were constantly occupied, and the East London Hospital contained 102 beds, 77 of which were constantly occupied Here the percentage of cost of administration to that of maintenance is 2½ per cent. more at Bristol than in London. The Glasgow Hospital for Sick Children, containing 70 beds constantly occupied, expended 8·858 per cent. upon administration, against 5·851 per cent. expended by the Evelina Hospital, which contains 66 beds, of which 51 were constantly occupied. If the Evelina be compared with the Edinburgh Hospital for Children (and these two institutions are about the same size), it will be seen that the London special hospital is managed at a cost of upwards of 13 per cent. less than that at Edinburgh. Our readers will be able to make other comparisons, but we have said enough to show that so far as the children's hospitals are concerned, those established in London for the most part expend materially less upon management than those situated in the provinces and elsewhere. We make bold to say that this state of things should be carefully considered by those immediately concerned, and we are confident, if the various committees decide to keep their books on the uniform system of accounts, a considerable reduction in the expenditure on management at the provincial and Scotch children's hospitals will speedily follow.

### Lying-in and Ophthalmic Hospitals.

Of course there are several other lying-in and ophthalmic hospitals to be found in the provinces, Scotland, and Ireland, besides the two included in the table. Many of them are, however, relatively small, and we have thought it best to narrow our inquiries on the present occasion to the two selected, because of their importance and size. The Rotunda Hospital, Dublin, is an ancient foundation of world-wide repute. It contains nearly double the number of beds to be found in any of the London lying-in institutions, and it treats considerably more than double the number of in-patients of any similar institution to be met with in the United Kingdom. Queen Charlotte's Lying-in Hospital, the largest in London, treated 972 in-patients during 1892, it had 38 beds constantly occupied, against 59 at the Rotunda Hospital, and expended 11·509 per cent. upon administration compared with 9·179 per cent. at the Rotunda, Dublin. These figures go to prove, as Queen Charlotte's expends more than any other metropolitan lying-in hospital upon administration, that when the increased accommodation at Dublin is allowed for, the cost of administering lying-in institutions is practically identical, whether the hospital be situated in Dublin or the metropolis.

Again, the Glasgow Eye Infirmary, with its 104 beds, of which 68 were constantly occupied, may be properly compared with the Royal London Ophthalmic Hospital in Moorfields, with its 100 beds, of which 83 were constantly occupied, the Glasgow institution having treated 1,326 in-patients, and 14,599 out-patients, against 1,967 in-patients and 26,495 out-patients treated at the Royal London Ophthalmic Hospital. The percentage of cost of administration to that of maintenance was 1·050 per cent. more at the Royal London than it was at the Glasgow Eye Infirmary, although the percentage of cost of administration to total ordinary expenditure at the former institution was only 10·667, compared with a percentage of 9·808 at Glasgow. These figures should prove eminently satisfactory to all concerned, but they strengthen and enforce the views we expressed in our article on "Ophthalmic Hospitals," published on September 30th, 1893, so far as those metropolitan ophthalmic hospitals are concerned which expend upwards of 20 per cent. every year upon administration. Looked at as a whole, we feel that the provincial, Scotch, and Irish special hospitals come out of the examination with credit, and that the figures in our table to-day enforce, strengthen, and support the percentages which we have ventured to lay down as a maximum, so far as expenditure upon administration is concerned.

No doubt, next year, when the various hospitals have had time to introduce the Uniform System of accounts, they will publish in their reports figures under all heads which may readily be compared with those of other institutions, because they will then relate to identical items of expenditure. The cost of administration in the best-managed hospitals will, we have every confidence, under the Uniform System of accounts, soon be found to be almost the same, wherever the buildings may be situated.

